# Cognitive Activity of an Individual Under Conditions of Information Influence of Different Modalities: Model and Experimental Research

**DOI:** 10.3390/e27030287

**Published:** 2025-03-10

**Authors:** Alexandr Y. Petukhov, Nikita S. Morozov, Nikolay V. Krasnitskiy, Yury V. Petukhov

**Affiliations:** 1 Information Security Center, Neimark University, 603138 Nizhny Novgorod, Russia; nvkrasnitskiy@gmail.com (N.V.K.); yuvpetukhov@yandex.ru (Y.V.P.); 2 Microelectronics Center, Neimark University, 603138 Nizhny Novgorod, Russia; nsmorozov@rf.unn.ru

**Keywords:** cognitive activity, virtual particles, information images, representations, Schrodinger equation, potential hole, self-oscillating quantum mechanics, 91E10

## Abstract

The objective of this study is to develop a mathematical model capable of correctly displaying the dynamics of an individual’s cognitive activity under conditions of external information influence of different modalities. The adequacy of the model is verified by comparing the results of numerical analysis and experimental data. The mathematical basis of such a model is the apparatus of self-oscillating quantum mechanics. To describe algorithms for the processes of transmission, processing, and generation of information, the theory of information images/representations is used. Methods. This article provides a brief description of the proposed theory. The cognitive activity of an individual is represented mathematically as a one-dimensional potential hole with finite walls of different sizes. The internal potential barrier in this model can model the boundary between consciousness and subconsciousness. The authors developed a parameterization of the systems under study taking into account the proposed theory. Next, a mathematical model was developed based on the apparatus of self-oscillatory quantum mechanics. The authors formulated an equation that describes the function of the state of an information image in the process of human cognitive activity. Computer modeling of various variations of information impact was carried out. The authors also conducted a specially designed experiment. Conclusions. The authors have identified characteristic patterns of such processes and shown the oscillating nature of changes in the state function of information images and the appearance of characteristic threshold effects. A comparison of the obtained model and experimental data showed the adequacy of the developed tool (the coincidence of the general dynamics and characteristic patterns was shown).

## 1. Introduction

Modeling the cognitive processes of the human brain is a very current topic. Scientists use a variety of mathematical tools to obtain new data and learn to predict specific cognitive processes. This task is very close to the topic of creating biosimilar artificial intelligence, which additionally attracts the attention of many research groups.

Nevertheless, efforts to define the mechanisms of information transfer and processing within an individual encounter a significant challenge at the core of contemporary cognitive science. The recent development of novel models of information transfer from an individual to another individual [1,2,3], processes of information processing in human memory [4,5,6], cognitive activity based on stochastic equations [7], classic “predator–prey” models [8], perception models [9,10], and others [11,12,13] represents a relatively recent phenomenon.

It must be acknowledged that a significant number of the aforementioned models are unable to provide a fundamental explanation of the processes of information transfer and its distortion caused by the interaction with the external communicative environment due to a lack of scalability and formalization.

Furthermore, a crucial aspect of human cognitive function is that the human mind does not operate in a manner analogous to a computer. The process of thinking occurs through the interaction of multiple images/representations. While a specific material foundation can be traced for these images, namely, electrical and chemical activity in the human brain, it is challenging to provide a comprehensive description of them in the form of a conventional mathematical model.

This article presents a new model of cognitive activity based on the theory of information images/representations, as well as elements of the mathematical apparatus of quantum physics [14]. The objective is to construct a model of an individual’s cognitive activity in the context of the impact of two distinct interacting information flows. These could, for instance, be a picture accompanied by sound, which can complement, contradict, enhance, or create the effect of cognitive dissonance. Verification of the developed model was achieved through a comparison of the results of computer simulation and experimental data (obtained on the basis of a specially designed experiment).

## 2. Brief Description of the Theory of Information Images/Representations

The proposed theory is based on the idea of a universal cognitive unit [11] of the information in the human mind—an information image (or representations), a space in which it exists, its topology and properties. Information images (hereinafter referred to as II) are the display of objects and events in any given feature space [15].

Accordingly, the theory of information images (hereinafter referred to as TII) can serve as a method to describe the exchange of information between individuals, as well as a number of cognitive functions of an individual.

The theory considers the human cognitive system as a structured set of interacting information images that are in constant dynamics and under the influence of many external and internal factors.

Images with higher energy (here, we would like to introduce a concept of l energy E to describe the communicative activity of II) are positioned “higher” and closer to the boundaries of the space of information images. These images interact with each other and the external environment more often than their low-energy counterparts. The latter are located closer to the center of space and relatively rarely enter into an active interaction with external stimuli. That is, the most active information images are our information portrait at the moment, what we are thinking about. The least active ones relate more to subconscious activity [15].

The TII provides a new look at a number of typical patterns in the human mind and contains tools for their proper interpretation and explanation.

Obviously, it is impossible to transmit one information image from one individual to another without any alterations. Each II is unique because each individual has their own specific set of individual experiences.

In addition, no one is capable of conveying an image that exists in their mind, their II space, to another person “as it is”, without any distortions. Such image transmission involves various communication devices that were shaped in our minds by a social superstructure of communication or the communication field (CF). The CF is the informational convergence of individual experience and collective unconscious formed as a result of an individual’s immersion into the social medium. The communication devices include speech, various visual, tactile, and symbolic methods, etc.

This article also proposes the following research hypothesis: an information object in the dynamics of cognitive processes is considered an object with physical properties.

There is no direct correspondence with a physical object here, but this view already differs from the classical approaches of Wiener and Shannon. Attempts at such consideration have already been introduced in a number of scientific studies; a number of philosophical theories and some theories for quantum physics (for example, “conformal field theory” and others that use virtual particles and quasiparticles to describe interactions) are closest to them. At the same time, these works introduce this either as a mathematical or as a philosophical abstraction. Based on analogs of the successful use of quasiparticles (which are essentially pure information objects, nevertheless described as physical), the use of the mathematical apparatus of quantum mechanics and its individual applications to describe information objects is proposed.

Accordingly, an object with physical properties must be described using the appropriate mathematical apparatus and tested on physical phenomena with similar dynamics. More about this can be found in previous works [16].

## 3. Mathematical Apparatus

In the framework of the model, the interaction of two individuals is presented as the interaction of two systems using communication fields (which are quantized by means of the IIs—as an analog of variable potential) in the information environment. Also of interest is the impact of a given information environment (for example, media, Internet resources, social environment, etc.) on an individual.

Accordingly, it is necessary to record the interaction function for these two individuals or the individual and the external influence.

We will then complicate the model by adding interactions between individual information images at the same energy level.

It is also important to note that the authors do not consider the human cognitive system as quantum, but only use the appropriate mathematical apparatus based on the analogy and logic of phenomenological approaches.

### 3.1. Self-Oscillatory Quantum Mechanics

There is a separate direction in quantum mechanics—self-oscillatory. It provides certain opportunities for solving problems that cannot be solved by conventional quantum mechanics [17]. One of the main properties of self-oscillating systems—a set of discrete energy states—is also a characteristic property of quantum systems. At the same time, conventional quantum mechanics does not consider the specific movement of an individual particle, so it cannot answer the question of whether it self-oscillates.

More information about this direction can be found in [17].

This direction is not generally accepted (although its results do not contradict classical quantum mechanics), but the most important thing is that, since we do not consider the human cognitive system as a quantum system, its exact correspondence to the quantum world is not fundamental for us. More important is how well this mathematical apparatus (and we will introduce a number of significant changes and a fundamentally different parameterization) will correspond to the description of cognitive processes. To check this, at the first stages there will be a comparison with real experimental and statistical data (not the author’s), and then, at later stages of this study, a full-fledged experimental study will be undertaken.

From the point of view of TII, the description of images/representations in the human mind in the form of an oscillatory system is quite convenient. It can reflect a number of characteristic properties of the studied effects and serve as an instrument for a more accurate study of the state of IIs as a result of internal interactions, movement, and overcoming barriers (i.e., information interaction with the environment). The frequency of oscillatory movements in the model for IIs can characterize the degree of “activity”, indicating the impact on the human mind from within. This expands the possibilities of a description of the model. In terms of classical quantum mechanics, it can be viewed as simply an energy level. In addition, this approach can help us to study the constructed state functions of an individual II.

### 3.2. Potential Pit Model

**Figure 1:**
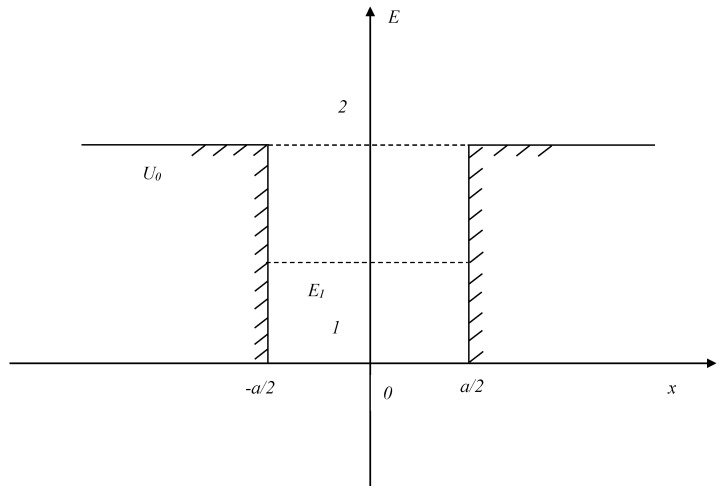
One-dimensional potential pit with finite walls for a self-oscillating case. Zone 1—inside the pit; Zone 2—outside.

Most of the mathematical apparatus has already been presented in the previous work, but we will partially repeat it in order to visually present the changes made for the calculations of the new problem. The kinetic energy of the particle inside the pit (Zone 1 in Figure 1) is as follows:

(1) $$E=\frac{mv_{1}^{2}}{2},$$

$$U\left(x\right)=\{\begin{array}{c} 0,-\frac{a}{2}<x<\frac{a}{2},\\ U_{0},\begin{array}{c} x\geq \frac{a}{2},\\ x\leq -\frac{a}{2} \end{array} \end{array}$$

Outside the pit:

(2) $$\frac{mv_{2}^{2}}{2}=E-U_{0}=-\left(U_{0}-E\right),$$

Hence,

$$v_{2}=i\left|v_{2}\right|,\;\left|v_{2}\right|=\sqrt{\frac{2}{m}\left(U_{0}-E\right)},$$

Next, we can write down the spatial parts of the wave equations for both zones at the specified “quantum” frequency (in our case, it is the frequency of the oscillatory movements of the IIs in the human mind). This value is directly related to the “activity” of the II:

$$\omega_{q}=\frac{2\pi mv_{1}^{2}}{h}=\frac{4\pi E}{h},$$

In Zone 1

$$\frac{d^{2}\varphi_{1}}{dx^{2}}+\frac{\omega_{q}^{2}}{v_{1}^{2}}\varphi_{1}=0,$$

Or

(3) $$\frac{d^{2}\varphi_{1}}{dx^{2}}+\frac{8\pi^{2}m}{h^{2}}E\varphi_{1}=0,$$

In Zone 2

$$\frac{d^{2}\varphi_{2}}{dx^{2}}+\frac{\omega_{q}^{2}}{v_{2}^{2}}\varphi_{2}=0,$$

Or

(4) $$\frac{d^{2}\varphi_{2}}{dx^{2}}-\frac{8\pi^{2}m}{h^{2}\left(U_{0}-E\right)}E\varphi_{2}=0,$$

$\varphi_{1,2}$ is a state function that satisfies the wave equation [17].

Accordingly, in Zone 1, the solution for the wave equation, and, in our case, the equation describing the state of the II, will have the following form [17]:

(5) $$\varphi_{1}=Asin\left(\frac{2\pi \sqrt{2mE}}{h}x\right),$$

In Zone 2 (outside the potential pit) it will have the following form:

(6) $$\varphi_{2}=Be^{\frac{2\pi E\sqrt{2m}}{h\sqrt{U_{0}-E}}x}+Ce^{-\frac{2\pi E\sqrt{2m}}{h\sqrt{U_{0}-E}}x},$$

At *x* > *a*/2, the coefficient B will be taken as equal to 0 (since, with the growth of *x,* it will increase to infinity, which is meaningless for the wave function), and, at a drop of *x* below –*a*/2, C will already be 0 [17].

What will the processes of interaction of this representation of the cognitive system look like in this case? First, it is necessary to describe the II beyond the boundaries of the cognitive system (potential pit). From a physical point of view, this occurs when the kinetic energy exceeds the required potential barrier. From the point of view of psychophysiology, this means that the image was activated in the human brain through the necessary electrical and chemical connections and was conditionally moved to the highest energy level corresponding to the current information activity.

Obviously, it will be possible to exit the II through the barrier if its energy exceeds the values of the barrier, i.e., from the point of view of the model, the velocity will look as follows:

$$v^{out}=v_{p}+\varepsilon ,$$

$v_{p}$—the velocity corresponding to the threshold (i.e., at which the kinetic energy is equal to the energy required for the barrier)

ε—any small increment of this speed, if necessary. Let us write down the equation of motion for the oscillatory system. In general, it looks as follows:

$$\frac{d^{2}x}{dt^{2}}=-\frac{k}{m}x,$$

where *k* is a certain coefficient characterizing the physical properties of the oscillatory system.

For a quantum system, this will look as follows:

$$\frac{d^{2}x}{dt^{2}}m=\mp \frac{\partial \varphi }{\partial x},$$

Since, according to (5) and taking into account the self-oscillatory movement $\cos\left(\omega_{q}t\right),\;\mathrm{the}\;\mathrm{following}\;\mathrm{is}\;\mathrm{true}$:

$$\varphi_{1}=Asin\left(\frac{2\pi \sqrt{2mE}}{h}x\right)\cos\left(\omega_{q}t\right),\;$$

Then, adding the possibility of the II going beyond the barrier, we will have the following form of the equation:

(7) $$m\frac{d^{2}x_{1}^{out}}{dt^{2}}=A\omega_{0}\sin\left(\omega_{0}x_{1}^{out}\right)\cos\left(\omega_{q}t+\beta \right)+\frac{2U_{0}}{a}\delta \left(\frac{2x_{1}^{out}}{a}-1\right),$$

where $\omega_{0}$ is the initial frequency of the oscillatory activity of the II.

β is the oscillatory motion phase.

δ is the Dirac function.

At values: −a/2 < x^out^ < a/2

(8) $$m\frac{d^{2}x_{2}^{out}}{dt^{2}}=C\frac{\omega_{q}}{\left|v_{2}\right|}e^{-\frac{\omega_{q}}{\left|v_{2}\right|}x_{2}^{out}}\cos\left(\omega_{q}t+\beta \right),$$

At values: *a*/2 < *x^out^*

(9) $$m\frac{d^{2}x_{3}^{out}}{dt^{2}}=B\frac{\omega_{q}}{\left|v_{3}\right|}e^{-\frac{\omega_{q}}{\left|v_{3}\right|}x_{3}^{out}}\cos\left(\omega_{q}t+\beta \right),$$

At values: *x_out_* < −a/2

Then, the equation of II state will look as follows:

(10) $$\varphi^{out}=\left(Acos\left(\frac{\omega_{q}x}{\sqrt{\frac{2}{m}E}}\right)|\frac{a}{2}>x_{out}+Ce^{-\frac{\omega_{q}}{\left|v_{2}\right|}x}|\frac{a}{2}<x_{out}\right)cos\omega_{q}t,$$

Here, we need to make some changes to the mathematical apparatus, which, of course, uses physical parameterization. We attempted to bring this parametrization to a stricter concordance with our problems, but still to a limited extent. Let us exclude $\omega_{q}$ from the equations keeping in mind that this is the “measure of activity” introduced by us for the II and that it directly depends on the energy of the individual IIs. So, let us rewrite it as follows:

(11) $$\varphi^{out}=\left(Acos\left(\frac{2\pi x\sqrt{2Em}}{h}\right)|\frac{a}{2}>x_{out}+Ce^{-\frac{4\pi E}{h\left|v_{2}\right|}x}|\frac{a}{2}<x_{out}\right)\cos\left(\frac{4\pi E}{h}t\right)\;,$$

Since we are not considering the process of external communication (outside the potential well in the quantum model) but rather internal processes in consciousness, the coefficient *C* in formula above will be zero. That is, IIs interact within an individual’s consciousness. However, in order to simulate the presence of two information images at the same energy level, it is necessary to introduce an interaction function:

$$D=He^{-4t^{2}/T^{2}}\cos\omega_{d}t$$

The activity of the image directly affects the amplitude of its oscillatory motion. In the case of growth exceeding the level of II’s activity/energy above the barrier, there is a regime change and going beyond the boundaries of the human mind (modeled by a potential pit), which corresponds to the communication process.

## 4. Experimental Study

Moreover, it is essential to evaluate the efficacy of the developed model in predicting real cognitive processes, particularly the interaction of two multimodal and multivalent images on an identical energy level. For this purpose, a bespoke experimental study was conducted [18].

A total of 227 people were selected as subjects: men and women from 17 to 58 years old. All subjects were examined to ensure they had no problems with cardiovascular, psychiatric, or respiratory diseases, and that they did not take any medications. All subjects were informed and signed an informed consent to participate in this study.

As stimulus material for the experiment, 24 images (12 extremely positive and 12 extremely negative according to the SAM valence scale) were selected from the international IAPS database and 24 sound stimuli (12 extremely positive and 12 extremely negative according to the SAM valence scale) were chosen from the international IADS database.

These visual and audio effective stimuli were randomly formed into specific blocks:

- Negative sound (S−) (12 negative sounds);
- Positive sound (S+) (12 positive sounds);
- Negative Image (P−) (12 negative images);
- Positive Image (P+) (12 positive images).

The subjects were asked to listen/watch the block and then evaluate it using the SAM technique.

In addition, four audiovisual stimulus sets were compiled from the selected stimuli:

- Negative video (image + sound) (P−S−) (12 negative sounds and 12 negative images);
- Positive video (image + sound) (P+S+) (12 positive sounds and 12 positive images);
- Dissonant video 1 (image + sound) (P−S+) (12 negative images and 12 positive sounds);
- Dissonance video 2 (image + sound) (P+S−) (12 positive images and 12 negative sounds).

These stimuli were grouped into the eight pairs of combinations/sessions presented in Table 1.

The exposure time was 6 s (in accordance with the playing time of one sound stimulus from the IADS database). After demonstrating each stimulus in combination, the subjects were assessed using the SAM method on three scales: valence, arousal, and dominance.

There was a pause of 20 min between the demonstrations of the first and second stimuli within the combination. The sessions were measured on different days to exclude the factor of interference from other stimuli on the individual’s emotional perception.

This study was conducted in a room isolated from external noise, with a comfortable workplace, in accordance with the basic requirements for conducting psychological testing.

**Figure 2:**
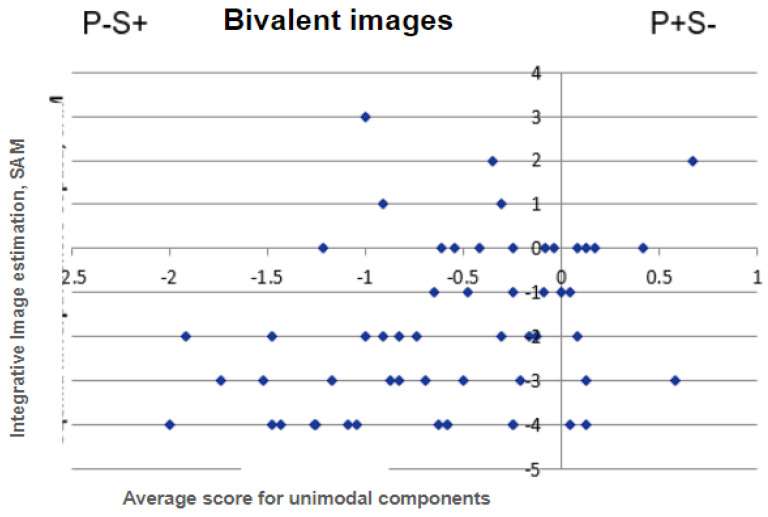
Phase space according to the integrative images SAM valence estimates with the subject’s average rating for unimodal components that were included in the integral bivalent videos.

The experiment demonstrated that, for a set of differently valenced stimuli, the dependence exhibits a pronounced non-linear character. Furthermore, the dominance of the negative image is also evident. The results of this experiment, as detailed in [18,19], are illustrated in Figure 2.

## 5. Results and Discussion

### 5.1. Simulation Results

The formula of external influence and its type and parameters are as follows:

$$D=He^{-4t^{2}/T^{2}}\cos\omega_{d}t$$

where *H* is the amplitude of the disturbance, *t* is the duration of exposure, *T* is the characteristic half-life, and ω_d_ is the natural frequency of exposure which we can consider as the main frequency with acceptable accuracy at this stage of development.

The final interaction of both information images, the second of which will act as an external influence, and the emergence of the final image (exit from the potential hole in the interpretation of the quantum model) can be observed as follows.

$$\varphi^{out}=\left(Acos\left(\frac{2\pi x\sqrt{2m\left(E+D\right)}}{h}\right)\right)\cos\left(\frac{4\pi E}{h}t\right)\;,$$

Next, we will consider several examples of how the model works with different parameters.

**Figure 3:**
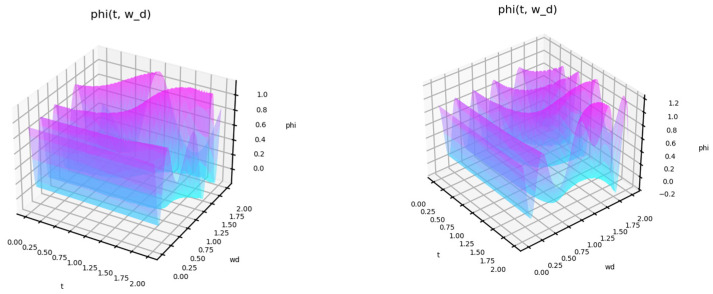
A status function for short-term exposure depending on the natural frequency of this exposure. Frequency w = 0.8, half-life T = 3 (**left**), T = 4.5 (**right**).

Figure 3 shows the type of state function for short-term exposure, depending on the natural frequency of this exposure. In Figure 3 (left), the half-life of the exposure is T = 3. In Figure 3 (right), T = 4.5.

**Figure 4:**
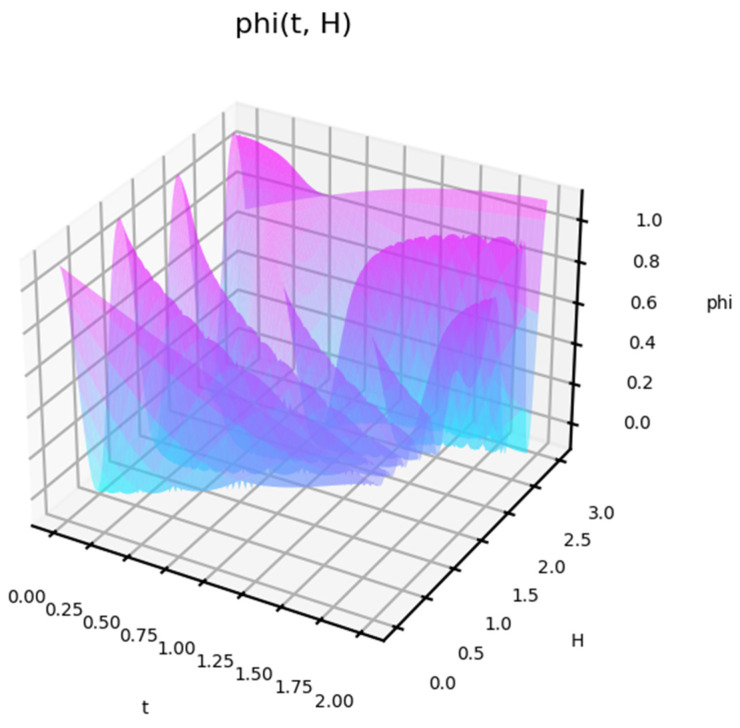
A status function for short-term exposure depending on the amplitude of this exposure. Frequency w = 0.8.

The behavior of the function shows how the response to different influences changes. The behavior of the function is nonlinear with time. For some frequencies, a fast response occurs first, but then a decline follows. The profile of this behavior is also non-linear. Figure 4 shows the state function depending on the amplitude of the impact.

**Figure 5:**
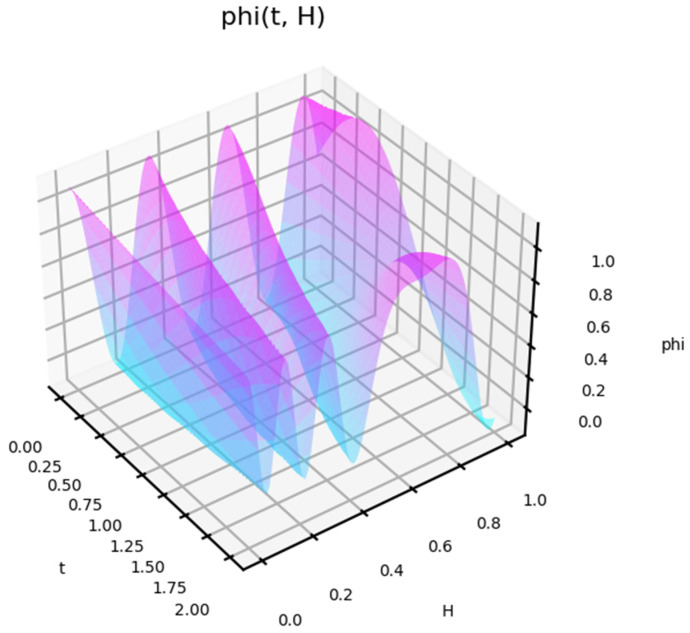
A status function for short-term exposure depending on the amplitude of this exposure. Frequency w = 0.8, T = 8.

This shows that, in triggering an impact response, its power is not a key factor. This is shown separately in Figure 5 for the case of a low-impact amplitude. The behavior in this case is also non-linear. For a small amplitude of the effect, the model shows cases of delayed (delayed) reaction.

Thus, we can observe a characteristic pattern of the appearance of nonlinear oscillating effects during the interaction of information images. This result is in good agreement with the data of existing experimental studies related to recording changes in the psychophysiological state of an individual under the influence of consistent and discordant external information factors (including multimodal ones) [20].

For example, it has been revealed and well tested (for example, in many horror films [21]) that complex combinations of images of different modalities and different “power” (from the point of view of their assessment by individuals separately on different bases) can give a more significant registered effect than, for example, a combination of unimodal images with maximum values across the bases. Alfred Hitchcock and many other authors demonstrate this very well with their films.

Thus, the impact effect quantitatively behaves nonlinearly, oscillating depending on a number of factors, having characteristic threshold effects and transition points, which were demonstrated numerically based on our model.

Now, let us check this in comparison with the data from our experiment.

### 5.2. Comparison of Results

It is crucial to highlight the specificity and non-linearity of the obtained characteristics. A comparison of the modeled data and the obtained experimental data is now presented. It is evident that the result of computer modeling exhibits a comparable character of distribution (a one-dimensional slice) at a specific set of parameters and reiterates the general characteristic regularities, as illustrated in Figure 4.

**Figure 6:**
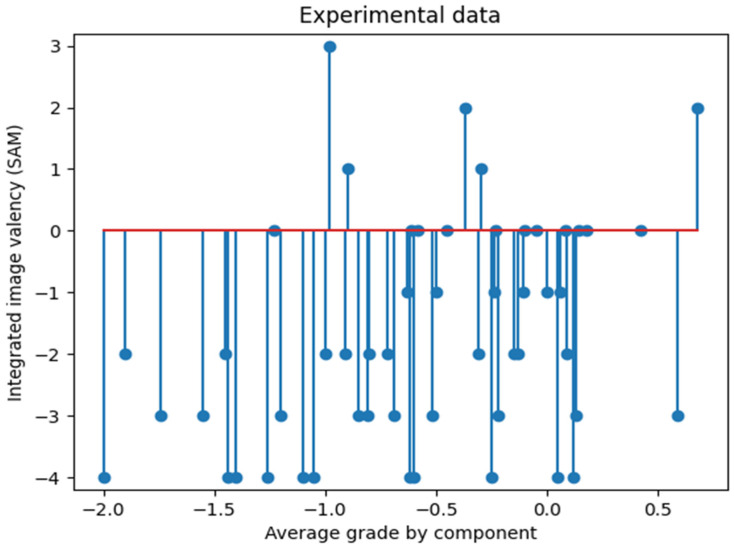
Experimental data of valence estimates under integrated exposure to multimodal images.

Consequently, by making a univariate slice of the multivariate function φ, we can obtain its form, which will correspond to the picture of the distribution of SAM valence scores of the integrative image of multimodal and multivalent stimuli seen in the experiment (Figure 6).

The selection of model parameters, specifically, frequency, amplitude, duration, and half-period of exposure, in manual mode is impractical due to the intricate nature and high sensitivity to minor oscillations in values. In order to fit the model, it is recommended that either analytical methods or optimization methods be employed.

**Figure 7:**
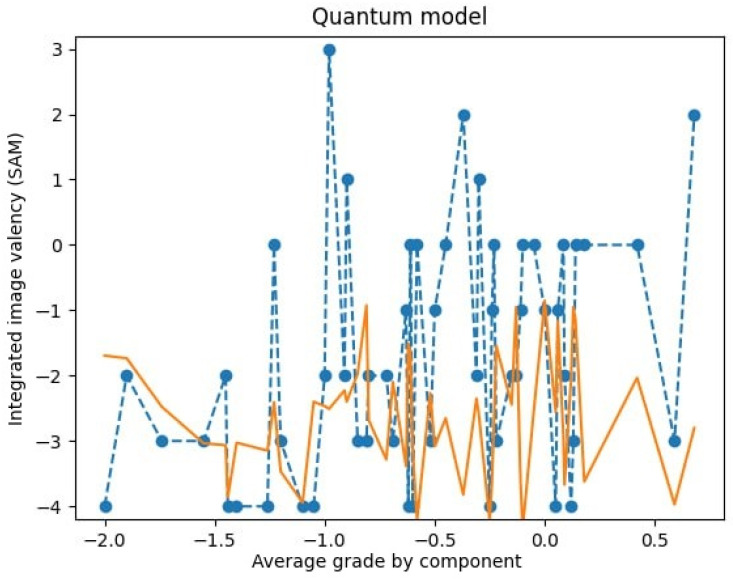
Experimental data (dashed line) and simulation results (solid line) with parameters fitted by the least squares method.

In the first iteration, the well-known method of least squares was used to select the parameters. The obtained result is shown in Figure 7.

It is noteworthy that, despite the inconsistency in magnitudes observed between the experimental data and the results obtained, the character of the oscillations is similar. It is evident that the relationships between the variables are markedly disparate. Consequently, the model in its current form is inadequate for accurately representing the experimental outcomes. A different set of parameters is necessary.

**Figure 8:**
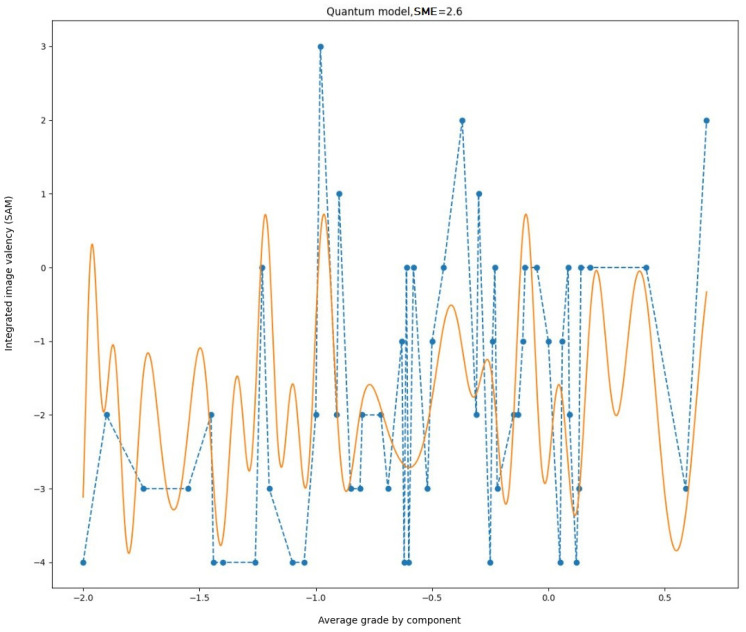
Experimental data (dashed line) and simulation results (solid line) with parameters fitted by the least squares method with other boundary conditions.

The subsequent stage was to augment the frequency and incorporate intermediate points. In other words, the boundary conditions and operating ranges of the parameters were modified. The outcome is presented in Figure 8.

It can be observed that the model has become more proficient at characterizing the quantitative oscillations observed in experimental data. However, in the domain of gradual initial value alteration, the emergence of beats that were not documented during the measurements suggests that the model remains inadequate and necessitates further enhancement.

**Figure 9:**
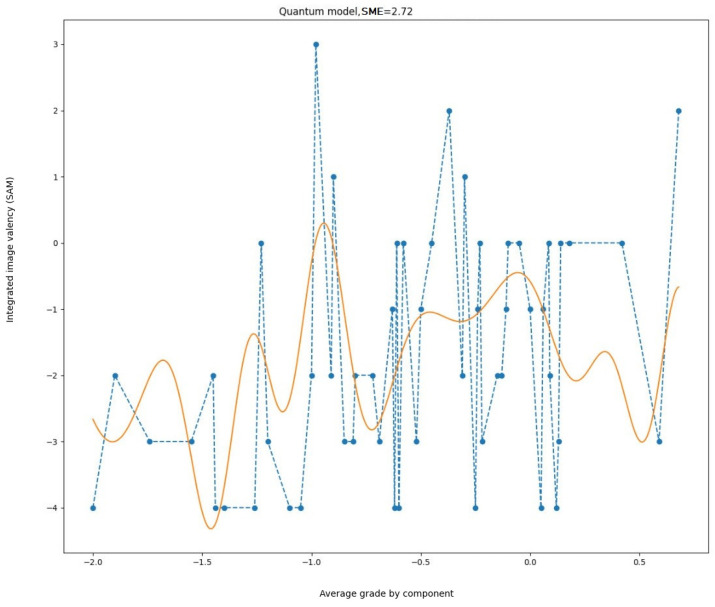
Experimental data (dashed line) and simulation results (solid line) with parameters selected by numerical method.

The method of parameter selection was modified. Rather than utilizing an analytical approach, a numerical method of multivariate optimization was employed. This is a considerably more costly process in terms of computational resources, but it can yield superior outcomes, as numerical methods permit the identification of the optimal (or locally optimal) point within a multidimensional parameter space without the necessity of constructing intricate analytical relationships. In this example, we used a search on a discrete grid of parameters with dynamically changing steps as we approach the local optimum. After a few attempts from different starting points chosen randomly, the most favorable result to date is illustrated in Figure 9.

The resulting graph demonstrates that the character aligns with the specified scaling factor. It should be noted that some values deviate from the expected outcome. They can be explained by the integrating property of the model itself when a small initial frequency of influence is taken as a point of departure. It is important to highlight that all parameters, which, in the quantum model, held a specific physical significance, are now merely conventional designations of various dimensionless parameters within a complex model of cognitive activity. Consequently, the term ‘frequency’ is also contingent and merely illustrates the nature of the influence exerted by this parameter of the model on the final outcome.

As demonstrated by the simulation results, the quantum model proposed and described in [16] is in accordance with the nature of the experimental data obtained in [18]. Further improvement requires the use of other numerical methods or, possibly, machine learning methods. One of the possible ways to improve the model is to supplement the discrete search with a simplex method. In this instance, it is feasible to augment the number of varying parameters by adding, for example, scaling coefficients by axes, which will facilitate a more precise alignment with the specific values of experimental data. An additional avenue for enhancement of the model is the extension of the search range of the optimal point within the multidimensional space of model parameters. At this phase, this was challenging to achieve due to the physical constraints of the hardware component of the simulation and insufficient optimization of the search algorithm itself. It is planned to supplement the model with an adaptive algorithm for selecting the range of variation of each parameter. Such an algorithm can be based on machine learning or on well-known adaptive filtering algorithms.

The results of this study can be used to predict the cognitive functions of an individual, which is useful, for example, in diagnosing cognitive disorders and constructing models for describing the activity of the human brain and systems with non-standard artificial intelligence.

## 6. Conclusions

Thus, in the framework of this study, we proposed a mathematical model based on the apparatus of self-oscillating quantum mechanics to describe the dynamics of information images within the framework of an individual’s cognitive activity.

On its basis, the authors modeled the interaction of two information images at the same energy level (i.e., the impact on an individual of two information influences of different or the same modality).

The authors have identified characteristic patterns of such processes and shown the oscillating nature of changes in the state function of information images and the appearance of characteristic threshold effects. Similar effects also appear in existing experimental data.

The authors also conducted a specially designed experiment. A comparison of the obtained model and experimental data showed the adequacy of the developed tool (the coincidence of the general dynamics and characteristic patterns was shown).

The next stage in the development of the research will be the refinement of the mathematical model. It is necessary to choose the correct range of parameter changes and correctly interpret their role in the formation and interaction of information images.

## Figures and Tables

**Table 1:** Incentive options.

№	Stimulus 1	Stimulus 2
1	P+	P+S−
2	P−	P−S+
3	S+	P−S+
4	S−	P+S−
5	P+S−	P+
6	P+S−	S−
7	P−S+	P−
8	P−S+	S+

## Data Availability

The data presented in this study are available on request from the corresponding author.

## References

[1] Kholodny Y.I., Malakhov D.G., Orlov V.A., Kartashov S.I., Aleksandrov Y.I., Kovalchuk M.V. (2021). The study of neurocognitive processes in the paradigm of information hiding. Exp. Psychol..

[2] Chernavsky D.S. (2009). Synergetics and Information. Dynamic Information Theory.

[3] Gevers W., Kadosh R.C., Notebaert W. (2011). Sequential Analysis of the Numerical Stroop Effect Reveals response suppression. J. Exp. Psychol. Learn. Mem. Cogn..

[4] Lee T.M., Liu H.L., Chan C.C., Ng Y.B., Fox P.T., Gao J.H. (2005). Neural correlates of feigned memory impairment. Neuroimage.

[5] Griffith D., Greitzer F.L. (2007). Neo-symbiosis: The next stage in the evolution of human-information interaction. Cogn. Inform. Nat. Intell..

[6] Vandekerckhove J. (2014). A cognitive latent variable model for the simultaneous analysis of behavioral and personality data. J. Math. Psychol..

[7] Faugeras O., Inglis J. (2015). Stochastic neural field equations: A rigorous footing. J. Math. Biol..

[8] Kooi B.W. (2015). Modeling the dynamics of traits involved in fighting-predators–prey system. J. Math. Biol..

[9] Haazebroek P., van Dantzig S., Hommel B. (2011). A computational model of perception and action for cognitive robotics. Cogn. Process..

[10] Velichkovsky B.B., Gusev A.N., Vinogradova V.F., Arbekova O.A. (2016). Cognitive control and sense of presence in virtual environments. Exp. Psychol..

[11] Anokhin K.V. (1989). Gene probes for mapping neural networks in learning. Principles and Mechanisms of Human Brain Activity.

[12] Pan F., Shi L., Lu Q., Wu X., Xue S., Li Q. (2016). The negative priming effect in cognitive conflict processing. Neurosci. Lett..

[13] Nikolaev A.R., Meghanathan R.N., van Leeuwen C. (2016). Combining. EEG and eye movement recording in free viewing: Pitfalls and possibilities. Brain Cogn..

[14] (1980). Physics of the Microcosm.

[15] Petukhov A.Y., Polevaya S.A. (2017). Modeling of cognitive brain activity through the Information Images Theory in terms of the bilingual Stroop test. Int. J. Biomath..

[16] Petukhov A.Y., Petukhov Y.V. (2022). Model of cognitive activity of the human brain based on the mathematical apparatus of self-oscillating quantum mechanics. Mathematics.

[17] Rodimov B.N. (2020). Self-Oscillatory Quantum Mechanics.

[18] Petukhov A.Y., Morozov N.S., Khaldina O., Krasnitskiy N.V., Polevaya S.A., Loskot I.V. The Impact of Multimodal and Polyvalent Audiovisual Stimuli on the Emotional State of an Individual. Proceedings of the 2024 Sixth International Conference Neurotechnologies and Neurointerfaces (CNN).

[19] Petukhov A.Y., Polevaya S.A., Polevaya A.V. (2022). Experimental Diagnostics of the Emotional State of Individuals Using External Stimuli and a Model of Neurocognitive Brain Activity. Diagnostics.

[20] Loskot I.V., Polevaya A.V., Polevaya S.A. Autonomic and cognitive displays of emotiogenic audiovisual stimuli. Proceedings of the 75th All-Russian School-Conference of Young Scientists with International Participation.

[21] Bogachev A.M. (2019). Horror films and nightmares: Some aspects of psychological analysis of phenomena. Psychol. Psychotech..

